# Comparative Evaluation of YOLOv8 and YOLO11 for Image-Based Classification of Sugar Beet Seed Treatment Levels

**DOI:** 10.3390/s26072137

**Published:** 2026-03-30

**Authors:** Cihan Unal, Ilkay Cinar, Zulfi Saripinar, Murat Koklu

**Affiliations:** 1 Department of Computer Programming, Hacettepe University, Sincan, Ankara 06909, Türkiye; cihan.unal@hacettepe.edu.tr; 2 Department of Computer Engineering, Selcuk University, Selcuklu, Konya 42130, Türkiye; mkoklu@selcuk.edu.tr; 3 Turkish Sugar Factories Corporation, Sugar Institute, Ankara 06810, Türkiye; zsaripinar@gmail.com

**Keywords:** deep learning, image classification, sugar beet seeds, YOLOv8, YOLO11

## Abstract

This study addresses the automatic classification of sugar beet seeds according to their spraying levels using RGB images, aiming to enable a fast, practical, and non-destructive early warning system without chemical analysis. A dataset of 16,519 seed images acquired under controlled lighting conditions was used to evaluate YOLOv8-CLS and YOLO11-CLS architectures, including the n, s, m, l, and x scale variants within the Ultralytics framework. All experiments were conducted using a 10-fold cross-validation strategy, with models trained under different batch size and learning rate configurations. The results indicate that both architectures achieve reliable performance, with accuracy values ranging from approximately 78–83% for YOLOv8-CLS and 80–82% for YOLO11-CLS models. ROC-AUC scores consistently above 0.94 demonstrate strong inter-class discrimination. Misclassification analysis shows that errors mainly occur between visually similar intermediate treatment levels, particularly 25% and 50%. Despite this challenge, low log-loss values and balanced precision–recall profiles indicate stable decision behavior. Overall, the findings confirm that sugar beet seed treatment levels can be effectively distinguished using only RGB imagery, providing a potentially low-cost and scalable approach for early warning and quality control in seed treatment processes.

## 1. Introduction

Seed quality is one of the fundamental factors directly affecting planting success, productivity, and product standardization in agricultural production. Seed treatment processes, in particular, are widely applied to provide protection against diseases and pests, but these processes also carry certain risks to the environment, workers, and human health. Therefore, in modern seed processing facilities, treatment processes are largely carried out through closed and controlled systems. However, mechanical failures, environmental conditions, changes in chemical properties, and application errors can lead to deviations in treatment doses and make it difficult to apply the targeted amount of pesticide to the seed surface.

Seed treatment applications involve the controlled application of biological and chemical agents to prevent seed-borne or soil-borne diseases, as well as pests that can damage seeds during germination and early development. The primary goal of this process is to ensure uniform, rapid, and healthy seed germination. Achieving adequate protection, particularly during germination and seedling stages, is directly related to the targeted dose and homogeneous distribution of the applied treatment across the seed surface [1,2].

Among the dosage errors encountered in practice, underdose application stands out both in terms of frequency and difficulty in detection. Under-dose applications can lead to inadequate seed protection, resulting in yield losses and plant health problems in the post-germination period. Current control approaches generally rely on chemical analysis of samples taken from treated seeds. However, these methods offer time-consuming, costly solutions that are not immediately applicable in every seed processing plant. Therefore, there is a growing need for a rapid, non-destructive, and practical control mechanism in the production line.

In the literature, seed quality assessment and classification problems have been addressed using classical image processing and machine learning approaches from RGB images, hyperspectral or multispectral imaging-based chemometric methods, and, more recently, deep learning-based architectures. Hyperspectral and multispectral imaging techniques, while offering high discrimination capability, have certain limitations in terms of hardware cost, setup complexity, and practical field applicability. Among deep learning-based approaches, real-time architectures such as the YOLO family have primarily focused on object detection and defect/quality analysis problems in agricultural and seed-related applications. Previous studies have successfully applied YOLO-based models to agricultural tasks such as seed defect detection, seed coating inspection, and quality analysis in maize and clover seeds [3,4,5]. Nevertheless, YOLO-based approaches may become less robust when inter-class differences are visually subtle, and larger variants may introduce higher computational cost without necessarily improving generalization under limited-data conditions [6,7,8]. Despite these advances, studies focusing on the direct classification of sugar beet seeds at incremental treatment levels (0%, 25%, 50%, and 100%), such as pesticide application rates, remain limited in the literature.

This study aims to address this gap by automatically classifying sugar beet seeds based on different spraying rates using RGB images. The proposed approach aims to reduce errors caused by human-based visual assessments and, in particular, to quickly detect errors caused by low-dose spraying. Thus, the goal is to create a decision support mechanism that can function as an early warning system in the production process without the need for chemical analysis.

In this context, the main contributions of the study can be summarized as follows:

- Classification of sugar beet seeds into four classes (0%, 25%, 50%, and 100%) based on their treatment rates using RGB images,
- Systematic comparison of the classification (YOLO-CLS) structures of the YOLOv8 and YOLO11 architectures across different model scales (n, s, m, l, x),
- Maintaining an accessible and low-cost display system while establishing a balance between model scale, accuracy, and practical usability,
- Evaluation of a generalizable early warning approach for seed treatment processes, as well as similar industrial processes involving color or coating applications.

Furthermore, while YOLO architectures in the literature are mostly used with a focus on object detection, this study prefers YOLO’s classification (YOLO-CLS) structure, adopting a simpler and task-specific learning approach that focuses directly on class differentiation rather than seed positioning.

## 2. Related Works

Automating seed quality and classification processes is critical for planting success, yield, and crop standardization. In the literature, this problem has been addressed using classical image processing and machine learning on RGB images, hyperspectral/multispectral imaging-based chemometric approaches, and deep learning-based methods, particularly those developed with real-time architectures like the YOLO family. This section summarizes studies conducted on different seed types in terms of data type, data size, methodological approaches, and performance metrics obtained.

### 2.1. Studies on Sugar Beet Seeds

Yang et al. aimed to predict the germination status of sugar beet seeds quickly and non-destructively using hyperspectral imaging, and reported high accuracies with SPA-selected wavelengths and models such as SVM-RBF on 3072 seeds [9]. Similarly, Zhou et al. compared different spectral preprocessing strategies on 3072 sugar beet seeds with VIS–NIR hyperspectral images; they obtained approximately 89% accuracy and 0.88 F1-score with LightGBM after characteristic wavelength selection [10].

In their 2018 study, Saripinar and Kesenci investigated the adhesion behavior of different physical forms of triple ready-mix formulations containing the same active ingredients on sugar beet seeds. The study compared the adhesion levels of hymexazole powder and liquid forms to the seed surface in formulations containing imidacloprid + thiram + hymexazole; it was reported that formulations using liquid hymexazole provided statistically significantly higher adhesion values compared to the powder form. High-pressure liquid chromatography (HPLC) was used to determine the amount of adhesion of the active ingredients on the seed. The findings revealed that in seed treatment, not only the active ingredient content but also the formulation type and application characteristics play a critical role in terms of dose accuracy and homogeneity [1].

Beyaz and Saripinar focused on the real-time detection/classification of coating defects in sugar beet seeds; they compared the YOLOv10-N/L/X models with 2000 high-resolution RGB images and reported the highest success with YOLOv10-X [3]. Salimi and Boelt addressed the classification of mechanical damages occurring during the polishing process in sugar beet seeds with multispectral imaging; they reported an average overall accuracy of 82% with nCDA on 19-band data based on VideometerLab [11].

### 2.2. YOLO-Based Seed Quality and Defect Detection

Xia et al. developed a YOLOv5-based approach for the automatic detection of surface defects in maize seeds; they showed that the model, improved with MobileNet backbone and ECA attention mechanism, is suitable and successful for portable applications [4]. Niu et al. also proposed an improved YOLOv8-based model for real-time detection of maize seed quality defects; they reported performance and speed gains with components such as EMA attention module, SPD-Conv and WIoUv3 [12]. For the recognition of coated seeds, Zhang et al. used a YOLOv5s-based model enhanced with CBAM in red clover seeds; they showed real-time usage potential in terms of mAP@0.5 and speed [5]. These studies reveal that the YOLO family offers the advantage of high accuracy and real-time in tasks such as seed quality/defect detection [3,4,5].

### 2.3. Hyperspectral and Multispectral Approaches

Hyperspectral imaging offers a powerful alternative, especially in scenarios where there are distinct spectral differences between classes, such as viability, maturity, and defects. Wang et al. investigated the maturity level in maize seeds using NIR-HSI; they obtained high accuracy values with different spectral representations and models [13]. Xu et al. addressed intrinsic/extrinsic defects in maize seeds with hyperspectral imaging; they reported high accuracy with a model that includes CNN (Convolutional Neural Network)-based feature selection and attention mechanism [14]. Similarly, Wang et al. classified the viability levels in waxy maize seeds using SVM (Support Vector Machine)-based models via hyperspectral data [15]. On the multispectral side, ElMasry et al. investigated multiple quality indicators such as senescence, germination, and seedling quality in fava bean seeds using LDA (Linear Discriminant Analysis)-based models with VideometerLab data [16]. In addition, Cui et al. aimed to quantitatively (regressively) predict the viability indicators in sweet maize seeds using hyperspectral data and evaluated KPCR-based models [17]. These studies show that spectral imaging approaches provide high discrimination; however, they may have some limitations in terms of equipment/installation cost and operational conditions [9,10,13,17].

### 2.4. Classical Machine Learning-Based Seed Classification

Koklu and Ozkan used size and shape features in the multi-class classification of dry bean seeds; they showed that SVM gave higher performance by comparing different classifiers [18]. For the classification of mechanical damage in flaxseed samples, Nadimi et al. compared both feature-based machine learning and transfer learning-based CNN models on X-ray images; they reported that the deep learning approach offered higher accuracy [19]. In addition, Koklu et al. showed that classical classifiers can achieve a certain level of success with morphological features in scenarios such as the differentiation of two varieties in pumpkin seeds [20].

In the case of the dry bean (13,611 samples) dataset [18], there are several studies in the literature comparing different preprocessing and modeling strategies on similar data. Khan et al. compared different models after outlier removal (IQR) and class adjustment (ADASYN); they reported that XGBoost gave the highest performance [21]. Macuacua et al. stated that data mining techniques such as PCA (Principal Component Analysis), hyperparameter optimization, and SMOTE improved performance, and that the KNN (K- Nearest Neighbors) model, in particular, achieved high accuracy [22]. These two studies were found to be representative in that they showed that classical ML (Machine Learning) approaches based on the same type of morphological features can be strengthened with different adjustment/optimization strategies; other studies with similar aims were considered complementary in this context [21,22].

The studies summarized above show that seed classification problems mostly focus on objectives such as defect/quality detection, variety differentiation, viability/maturity estimation, or coating/processing damage analysis [4,17]. Although hyperspectral and multispectral approaches offer high discrimination, they may have limitations in terms of equipment cost and field applicability [9,10,11,13,14,15,16,17]. A significant portion of YOLO-based studies focus on object detection and defect/quality assessment; direct classification of sugar beet seeds according to graded levels, such as treatment rate (0%, 25%, 50%, 100%), is addressed only to a limited extent in the literature [3,4,5,12]. In this study, the aim was to classify sugar beet seeds into four classes according to their treatment rates using RGB images; YOLOv8 and YOLO11 classification models were systematically trained with n–s–m–l–x variants and compared. Thus, a balance between accuracy and practical usability of different model scales has been demonstrated while maintaining an accessible image layout (RGB). Furthermore, while YOLO architectures in the literature are mostly used with a focus on object detection, this study prefers YOLO’s classification (YOLO-CLS) structure, adopting a simpler and task-specific learning approach that focuses directly on class differentiation instead of seed placement.

## 3. Materials and Methods

**Figure 1:**
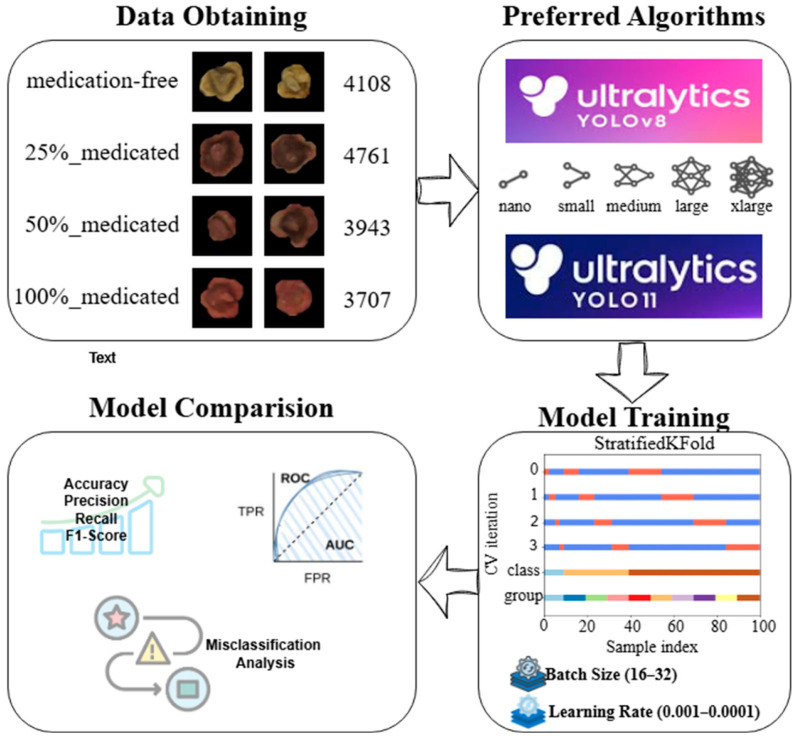
Flowchart of the study methodology.

This section details the dataset and imaging layout created from RGB images representing four different treatment levels (0%, 25%, 50%, and 100%) of sugar beet seeds, the experimental design for training the YOLOv8-CLS and YOLO11-CLS models with different scale variants (n–s–m–l–x), and the metrics used to evaluate performance. The flowchart of the study methodology is given in Figure 1.

### 3.1. Dataset Description

In this study, a unique RGB image dataset was created for classifying the pesticide application dose levels of sugar beet seeds based on image data. The dataset consists of four classes according to the application level: medication-free (0%), 25%_medicated (25%), 50%_medicated (50%), and 100%_medicated (100%). These treatment levels were selected to represent the practically meaningful range of application conditions in seed treatment processes: 0% as the untreated reference, 100% as the nominal/full treatment level, and 25% and 50% as intermediate under-dose levels that may occur in practice due to application inconsistencies or system-related deviations. Since the seed samples were obtained in separate batches with known application status, the labeling process was carried out by capturing images separately for each class during the shooting process and saving them to the relevant folder without requiring manual annotation. Thus, “ground truth” information was reliably established based on the class information from the source of the seeds.

The dataset contains a total of 16,519 images. The sample sizes by class are 4108 for medication-free, 4761 for 25%_medicated, 3943 for 50%_medicated, and 3707 for 100%_medicated, respectively. During dataset preparation, no images were removed or excluded based on visual quality criteria such as blur, illumination variation, or partial occlusion. All captured images were retained to preserve the natural variability of the acquisition conditions and to avoid introducing selection bias. Importantly, each image corresponds to a distinct physical seed and no multiple captures of the same seed were included in the dataset.

**Figure 2:**
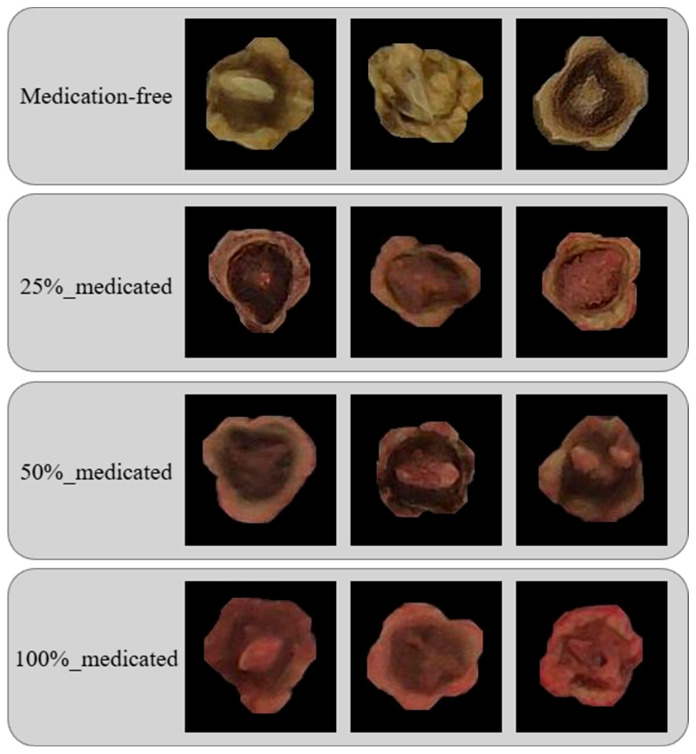
Example images for each class in the dataset.

The images were visually inspected only to ensure that the seed was visible within the frame and that the file was not corrupted. Apart from these minimal checks, no additional filtering was applied. This approach ensures that the dataset reflects realistic acquisition conditions and avoids potential bias introduced by selective image removal. Sample images for each class in the dataset are given in Figure 2.

**Figure 3:**
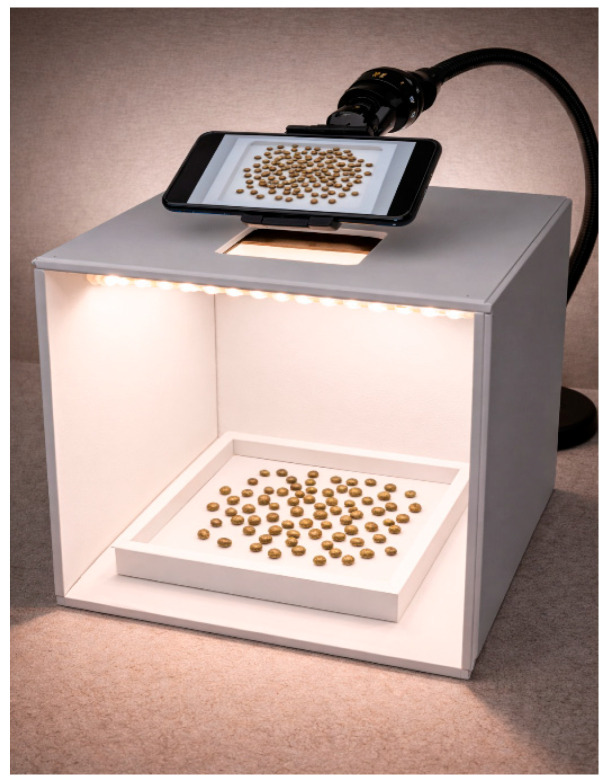
Schematic representation of the controlled image acquisition setup. (This image was generated using an AI-assisted tool for illustrative purposes and does not represent a raw experimental photograph).

The same imaging protocol was followed for all classes during the data acquisition process. Seed images were recorded using a Xiaomi 11T Pro smartphone camera under constant illumination in a shooting box isolated from ambient light. The illumination was provided by a strip LED with a color temperature of approximately 4000 K, and the light intensity was fixed at approximately 1150 lx, measured in the shooting plane (the nominal luminous flux of the lighting used was approximately 500 lm). With this setup, the aim was to minimize variations in light intensity, shadow formation, and reflection-induced variability, thereby increasing visual comparability between classes. During imaging, seeds belonging to each treatment level were handled separately, and after completing the shots under the same setup conditions, the images were directly transferred to the relevant class folders. The treatment levels used as class labels were determined from controlled seed-treatment batches with predefined application rates. Production records documenting the applied treatment levels were used as the reference for labeling. During image acquisition, seeds originating from each treatment batch were photographed separately and stored in class-specific directories corresponding to their known treatment level. This approach strengthens the repeatability of the data collection process while providing a more stable input structure for model training. A representative image of the data acquisition process is given in Figure 3.

### 3.2. Model Architectures

The YOLO (You Only Look Once) family of single-stage deep learning architectures is known for offering a balance between speed and performance, particularly in real-time computer vision applications. The general structure of YOLO architectures consists of a backbone that learns multi-level representations from images, a neck that combines these representations to produce richer features, and a head that generates task-specific output. This modular structure enables the capture of multi-scale visual cues and allows for adjustment of the capacity-computational cost balance according to different application requirements [7].

Although YOLO is mostly used for object detection in the literature, YOLO-CLS versions are offered for classification problems in the Ultralytics (version 8.3.158) ecosystem. In detection-based YOLOs, the model combines multi-level features obtained from the backbone in a multi-scale manner via the neck and produces class labels with bounding box predictions with the detection head. In contrast, the goal of YOLO-CLS versions is not to find the object’s location, but to classify the image directly. Therefore, bounding box regression and components for the detection head are not used. The feature representation learned by the backbone is transferred to the classification head, and class probabilities are produced as output [7,8,23,24]. In this study, since the seeds are clearly positioned in the center of the image and there is no need for object positioning, YOLOv8-CLS and YOLO11-CLS models, which do not require bbox annotation and offer a simpler learning structure, were preferred.

The reason these models were preferred is that the YOLO-CLS architectures directly supported for classification tasks within the Ultralytics ecosystem are limited to the stable and widely used YOLOv8-CLS and YOLO11-CLS versions at the time of the study. The more recent YOLOv12 architecture was not included in the experimental scope of this study because it does not yet offer a stable and widely validated version specifically for classification.

**Figure 4:**
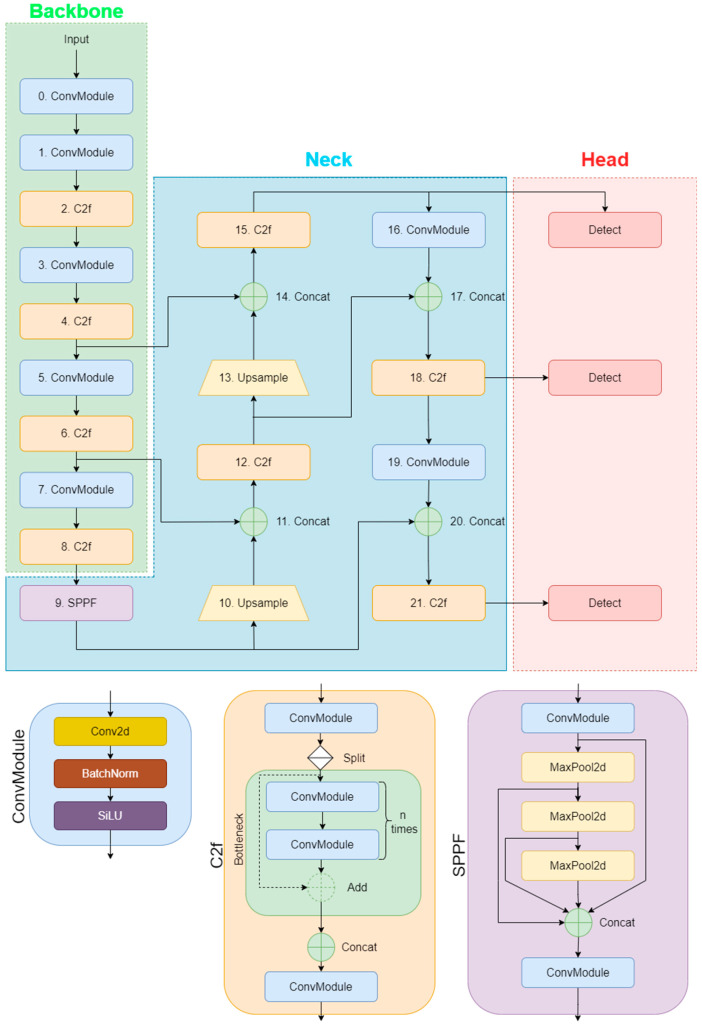
The backbone–neck–head components and core modules of the YOLOv8 detection architecture.

**Figure 5:**
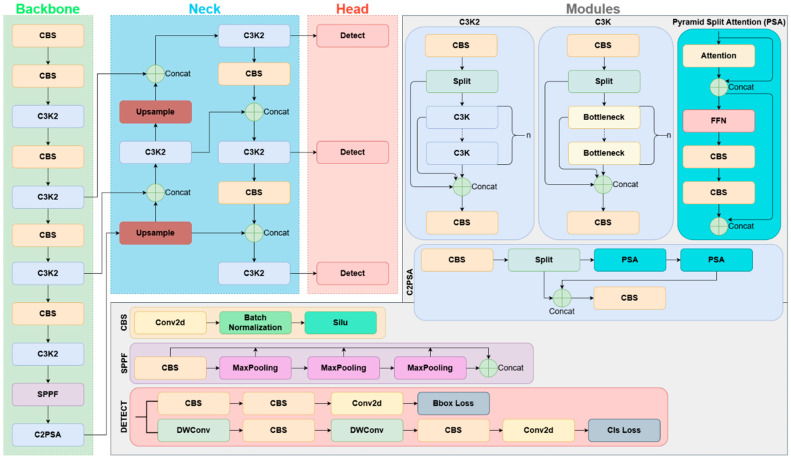
The backbone-neck-head components and core modules of the YOLO11 detection architecture.

YOLOv8 is an architecture widely used by Ultralytics and offers a strong foundation with optimized block designs. YOLO11, a more recent version within the same ecosystem, includes architecture/implementation-level updates aimed at improving the balance between representation capacity and efficiency. The joint evaluation of these two versions aims to experimentally determine which version and which scale variant is more suitable for discriminating subtle visual differences, such as pesticide application levels, in sugar beet seeds [6,23,24]. Schematic representations of the YOLOv8 and YOLO11 architectures are presented in Figure 4 and Figure 5.

Both model families were initialized with pre-trained classification weights (e.g., yolov8n-cls.pt, yolo11s-cls.pt) provided by Ultralytics. Within the scope of transfer learning, the final classification layer of the models was restructured into 4 classes to match the target number of classes for the study. Thus, while general visual representations learned in the early layers were preserved, the final layers were adapted to learn distinctions specific to the treatment levels of sugar beet seeds.

YOLO-CLS models are presented with n, s, m, l, and x scale variants. As the scale increases, the number of parameters and representation capacity increase, which can improve performance, especially in problems where visual differences between classes are limited. However, larger scales lead to higher computational costs and longer inference times [7,8]. Therefore, in this study, not only the highest accuracy but also the accuracy-efficiency balance, which is critical for practical use, was examined by systematically training and comparing all scales for both architectures.

### 3.3. Training Strategy

In this study, a 10-fold stratified cross-validation (StratifiedKFold) approach was adopted for training the YOLOv8-CLS and YOLO11-CLS models to prevent results from being tied to a single training or validation tier and to increase performance. The stratified tier approach ensures the preservation of class ratios in each fold, reducing bias that may arise from class distribution. Accordingly, the dataset was divided into 10 folds, nine tiers were used for training and one tier for validation in each fold, and when all folds were completed, performance metrics were reported on a fold basis, and general results were obtained from average values [25,26].

Each image in the dataset corresponds to a unique physical seed, and no repeated captures of the same seed were included. Consequently, the dataset does not contain near-duplicate samples originating from the same object. The stratified 10-fold cross-validation therefore operates on independent seed instances, reducing the risk of information leakage between training and validation folds.

During the training process, model weights were restarted for each model/scale configuration (while maintaining the transfer learning start) and independently trained, with the same training protocol applied to each fold. Input images were rescaled to 224 × 224 and fed into the model in RGB tensor format to ensure consistency across all experiments. Cross-entropy loss was used as the loss function for the multi-class classification problem. The Adam algorithm was preferred in the optimization phase, and two different learning rate combinations (1 × 10^−3^ and 1 × 10^−4^) and two different batch size combinations (16 and 32) were tested to systematically examine the effect of hyperparameters such as learning rate and batch size on performance. Each training session was run for a maximum of 30 epochs.

An early stopping strategy was implemented to limit overfitting and efficiently manage training time. The early stopping mechanism works by monitoring the improvement in validation performance, the counter is incremented when validation accuracy does not improve above a certain threshold value (min_delta = 5 × 10^−4^), and training is terminated if no improvement is observed over 7 consecutive epochs. Thus, the best model for each fold was determined as the weights in the epoch that maximized validation accuracy, and these weights were recorded separately. The final metrics and analyses were calculated based on this best model selected for each fold.

No separate external hold-out test set was used in this study. Instead, model generalization was assessed through stratified 10-fold cross-validation, and potential overfitting was monitored by early stopping based on validation accuracy, together with fold-wise consistency of accuracy, F1-score, log-loss, and ROC-AUC values.

To examine the model’s decision-making behavior in more detail, misclassifications were recorded as part of the error analysis at the end of training. These analysis steps aim to support performance not only with numerical metrics but also by examining which visual cues the model relies on, as part of the training strategy.

### 3.4. Evaluation Metrics

This study used multiple performance metrics to evaluate model performance not only based on the correct or incorrect ratio, but also to more comprehensively assess inter-fold interference and the model’s predictive confidence. All metrics were calculated separately for each fold within a 10-fold stratified cross-validation framework, and the final results were reported by averaging the fold-based values. In the calculations, model outputs were converted into probabilities using the softmax function, and class labels were obtained by selecting the class with the highest probability.

The confusion matrix is one of the most important diagnostic tools that complements the metrics. By showing which class each true class is predicted to be at the cell level, the confusion matrix reveals which classes the model systematically confuses. From this matrix, the number of true positives (TP), false positives (FP), true negatives (TN), and false negatives (FN) can be derived for a specific class. TP represents the examples that belong to the relevant class and are correctly predicted, FP represents the examples that do not belong to the relevant class but are predicted to be that class, FN represents the examples that belong to the relevant class but are predicted to be another class, and TN represents all other true negative examples [27,28,29]. In this study, a confusion matrix was generated for each fold; furthermore, the matrices obtained across the folds were combined to calculate the average confusion matrix, which was then presented as a percentage using row normalization. This allowed for a clearer analysis of the confusion trends between spraying levels.

Accuracy is one of the most fundamental performance metrics in classification problems and expresses the proportion of correctly classified samples among all samples. It can be interpreted as a general indicator of success, but it may not be sufficient on its own in cases where the sample sizes between classes are unequal, or some classes are visually closer to each other [27,28,30]. In multi-class classification problems, accuracy is defined as the ratio of the number of correctly predicted samples for all classes to the total number of samples. In other words, the accuracy metric summarizes the average classification success of the model under a single measure without evaluating each class separately. Therefore, accuracy was used as the first indicator of overall performance in this study, but the evaluation was supported by other metrics. The formula for calculating accuracy is given in Equation (1)

(1) $$Accuracy=\frac{\sum_{i=1}^{K}{TP}_{i}}{N}$$

Here, K: Number of classes, ${TP}_{i}$ the number of correctly classified samples for class i, N represents the total number of samples.

Precision indicates how many of the samples that the model predicts for a particular class actually belong to that class. In other words, precision reflects the effect of false positive predictions and provides information about “how reliable the model is when it says that class”. When spraying levels are close to each other, for example, when classes are mixed such as 25% and 50%, the precision value helps to understand which classes the model gives more false alarms for [27,28,30]. The precision calculation formula is given in Equation (2)

(2) $${Precision}_{\left(weighted\right)}=\frac{\sum_{i=1}^{K}n_{i}\;P_{i}}{\sum_{i=1}^{K}n_{i}}$$

Here, K: represents the number of classes, and n_i represents the number of supports for class i.

Recall (Sensitivity) refers to how many real instances of a particular class are correctly captured by the model. Recall is sensitive to false negative errors and reveals the model’s tendency to miss the relevant class. Since the goal of this study can also be adapted to early warning logic (e.g., not missing a low dose), the recall metric is particularly important in evaluating the model’s coverage, especially for critical classes [27,28,30]. The recall calculation formula is given in Equation (3)

(3) $${Recall}_{\left(weighted\right)}=\frac{\sum_{i=1}^{K}n_{i}\;R_{i}}{\sum_{i=1}^{K}n_{i}}$$

Here, K: represents the number of classes, and n_i represents the number of supports for class i.

The F1-score is the harmonic mean of the precision and recall metrics and presents these two metrics balanced under a single number. In cases where one precision and recall may increase while the other decreases, the F1-score provides a more holistic measure of balanced performance. In this study, the F1-score was used to more robustly summarize the overall model quality, especially in a multi-class scenario where inter-class interference is present [27,28,30]. The formula for calculating the F1-score is given in Equation (4)

(4) $$F1-score=2\times \frac{Precision\times Recall}{Precision+Recall}$$

Since these metrics are calculated on a class-by-class basis in multi-class problems, weighted average values are also reported in the study. The weighted average approach weights the contribution of each class by the number of samples belonging to that class, allowing for a summary of overall performance consistent with the data distribution. Thus, while the impact of classes with relatively more samples on total performance is naturally represented, an overall view of class-based performance is obtained.

The Log-loss (Cross-Entropy Loss/Negative Log-Probability) metric was calculated to evaluate not only the class labels of the model but also the quality of the probabilities it generates. Since Log-loss penalizes situations like assigning a lower probability to the correct class with a higher penalty, it provides information about whether the model’s prediction confidence (calibration) is good. For example, even if two models have the same accuracy value, if one systematically assigns a higher probability to the correct class, the log-loss value will be better (lower). Therefore, log-loss is considered a more finely tuned performance indicator compared to accuracy [31,32]. The formula for calculating the Log-loss is given in Equation (5)

(5) $$L=\frac{1}{N}\sum_{i=1}^{N}\sum_{c=1}^{C}y_{i,c}\log\left(p_{i,c}\right)$$

Here, N represents the number of samples, $y_{i}\in \;\left\{0,\;1\right\}$ is the true label, and $p_{i}$ is the predicted probability of class 1.

Finally, ROC curve and AUC (Receiver Operating Characteristic and Area Under the Curve) analysis were performed to evaluate the discrimination power in multi-class classification. The ROC curve shows the change between the true positive rate and the false positive rate at different decision thresholds and allows the model’s capacity to separate classes independently of the threshold [31,32]. In the multi-class problem, ROC/AUC calculation was performed separately for each class using a one-vs-rest approach. The macro-AUC was reported by averaging the AUC value of each class. This approach summarizes the overall discrimination of the model by treating the classes with equal importance. To ensure comparability between folds, the ROC curves were interpolated onto a common FPR grid and an average ROC curve was created. Thanks to this set of metrics, the models were comprehensively evaluated not only in terms of overall accuracy but also in terms of class-based error types, reliability of probability estimates, and threshold-independent discrimination level.

### 3.5. Implementation Details and Hardware

All experiments were conducted using a Python-based experimental framework. The YOLOv8-CLS and YOLO11-CLS models were used via the Ultralytics library; training and evaluation were carried out using PyTorch. Cross-validation partitions were created using scikit-learn (StratifiedKFold). Classification reports, confusion matrix, ROC-AUC, and log-loss calculations were obtained using scikit-learn metrics. Standard Python visualization tools were used for visualization and recording of outputs.

Experiments were conducted on a system featuring an NVIDIA GeForce RTX 4060 Ti (8 GB) graphics card (NVIDIA, Santa Clara, CA, USA), an Intel i7-12700K processor (Intel, Santa Clara, CA, USA), and 64 GB of RAM. This hardware configuration enabled the training of different model scales (n–s–m–l–x) under 10-fold cross-validation, while also providing a basis for cost evaluation based on model scale in practical use cases.

For reproducibility, the key training hyperparameters used in the experiments are summarized as follows: input resolution of 224 × 224, Adam optimizer, learning rates of 1 × 10^−3^ and 1 × 10^−4^, batch sizes of 16 and 32, maximum training length of 30 epochs, and early stopping with a patience of 7 epochs (min_delta = 5 × 10^−4^). All experiments were conducted using stratified 10-fold cross-validation with identical training protocols across folds.

## 4. Results

### 4.1. Overall Classification Performance

Different batch size and learning rate combinations were tested for each model scale; all combinations are presented in the tables, and the results providing the highest performance for the relevant scale are taken as the basis for the interpretations. Quantitative results for the YOLOv8-CLS and YOLO11-CLS models are summarized in Table 1 and Table 2, respectively.

When examining YOLOv8-CLS-based models, the highest classification performance is observed in the small-scale YOLOv8-n variant. This model consistently produced the highest accuracy, weighted F1-score, and ROC-AUC values under different training configurations. In the larger-scale YOLOv8-m, l, and x variants, a limited decrease in performance was observed. This suggests that the limited dataset size and inter-class visual differences may negatively impact performance reliability in higher-parameter models. However, the high ROC-AUC values (approximately 0.94–0.95) in all YOLOv8 variants indicate that the models have largely learned the distinguishing visual cues between spraying levels. The similarity of weighted precision, recall, and F1-score values suggests that the models exhibit stable classification behavior despite the imbalance in class distribution. Furthermore, relatively low log-loss values indicate consistent probability estimations.

YOLO11-CLS architectures are generally comparable to YOLOv8-CLS models, exhibiting a more balanced performance profile at some scales. In particular, accuracy and weighted F1-score values remain stable in small and medium-sized YOLO11 variants, while ROC-AUC metrics maintain their high levels. The low log-loss values in YOLO11 models support the idea that the results are not due to overfitting and that the model outputs are reliable. However, it has been observed that performance improvement is limited as the scale increases in YOLO11 architectures as well. This is related to the nature of the classification problem and the scale of the dataset.

Both architectures offer satisfactory and reliable performance in the problem of classifying RGB images according to spraying levels. The results show that model scale alone is not the sole determinant of performance; rather, selecting a capacity compatible with the dataset characteristics is more critical. In light of these findings, the effect of model scale (n–s–m–l–x) on classification performance is analyzed in detail in the next section.

### 4.2. The Effect of Model Scale (n–s–m–l–x) on Classification Performance

In YOLO architectures, the model scale (n, s, m, l, and x) directly affects the model’s representational capacity by determining the number of layers in the backbone and classification header, as well as the channel widths. As the scale increases, the number of parameters and the variety of complex patterns the model can learn also increase, but conversely, computational cost and the risk of overfitting also rise. Therefore, systematically examining the impact of different scales on performance is critical for both model selection and practical application scenarios.

The results obtained in this study show that model scale does not have a one-way increase effect on classification performance. The highest accuracy and weighted F1-score values in the YOLOv8-CLS architecture were obtained in the small-scale YOLOv8-n variant, revealing that larger models do not always offer better performance. The limited decrease in accuracy values in the medium and large-scale YOLOv8-m, l, and x models suggests that dataset size and visual differences between spraying levels may not be sufficient to fully utilize the potential of high-capacity models. This highlights the importance of the balance between model capacity and data complexity in classification problems.

A similar trend has been observed in YOLO11-CLS architectures. Small and medium-sized YOLO11 variants exhibit stable performance in accuracy and weighted F1-score metrics, while gains are limited as scale increases. These findings suggest that, despite the more current representation learning capabilities of the YOLO11 architecture, excessively large models do not provide an additional advantage due to the problem scale and data structure. The fact that differences in spraying levels are often based on subtle visual cues such as color intensity and coating homogeneity suggests that more compact models may be advantageous in terms of better performance on unseen data for such problems.

Although YOLO11 incorporates more recent architectural refinements compared to YOLOv8, such improvements do not necessarily guarantee superior performance on every dataset. In the present study, the classification task relies primarily on subtle visual cues related to seed coating intensity under controlled imaging conditions. Under such circumstances, compact architectures with fewer parameters may generalize slightly better, particularly when the dataset size is moderate and the discriminative cues are mainly color-based. Consequently, the lightweight YOLOv8-n configuration achieved marginally higher performance than several larger or newer variants.

When the effect of model scale is evaluated through ROC-AUC metrics, maintaining high ROC-AUC values at all scales indicates that the models generally learned to successfully differentiate between classes. However, scale-dependent differences in accuracy and F1-score metrics suggest that confounding, particularly between adjacent spraying levels (such as 25% and 50%), stems from visual similarities between classes rather than model capacity. This reveals that increasing the model scale alone is not sufficient to completely eliminate such confounding.

The findings indicate that model scale selection should be problem-oriented. For problems with a limited number of classes and relatively subtle visual differences, such as the classification of sugar beet seeds according to their treatment levels, small and medium-sized YOLO-CLS models offer a more balanced solution in terms of both high accuracy and lower computational cost. This is considered a significant advantage, increasing the potential applicability of the proposed approach in practical monitoring scenarios.

### 4.3. Class-Wise Performance and Error Analysis

This section provides a detailed analysis of the class-based behavior of the top-performing YOLOv8-CLS and YOLO11-CLS models, using confusion matrices and misclassified examples. This analysis aims to go beyond simply assessing overall accuracy and to reveal error tendencies stemming from visual similarities between the models’ spraying levels.

#### 4.3.1. Confusion Matrix Analysis

**Figure 6:**
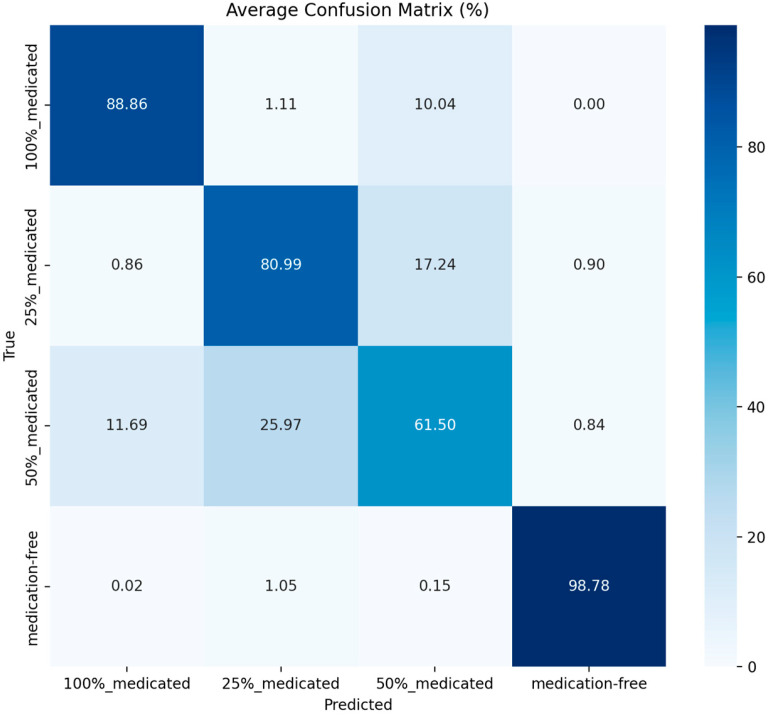
Average confusion matrix for YOLOv8-n (batch = 32, lr = 0.001).

**Figure 7:**
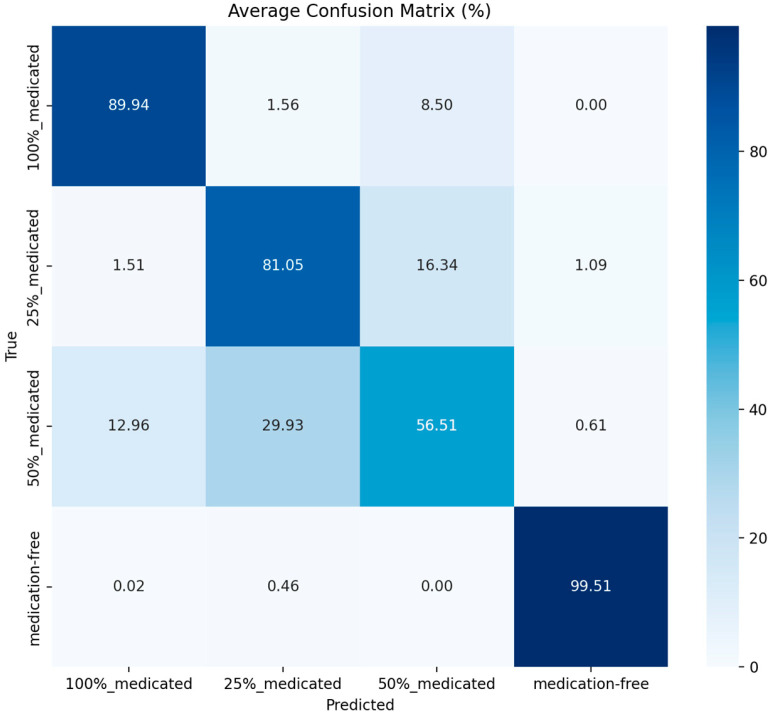
Average confusion matrix for YOLO11-n (batch = 32, lr = 0.0001).

The confusion matrices obtained during 10-fold cross-validation for the YOLOv8-n (batch size = 32, learning rate = 0.001) and YOLO11-n (batch size = 32, learning rate = 0.0001) models were centered and row-normalized, and are presented as percentages (%) in Figure 6 and Figure 7.

Analysis of the confusion matrices reveals that both models show the medication-free class has the highest classification accuracy. The correct classification rate for this class is 98.78% in the YOLOv8-n model, while it increases to 99.51% in the YOLO11-n model. This indicates that medication-free seeds are more distinctly differentiated from treated classes in terms of visual characteristics such as color, surface texture, and gloss.

A significant mixing tendency was observed between the 25% and 50% treated classes. In the YOLOv8-n model, approximately 25.97% of the 50% treated seeds were classified as 25% treated, and similarly, 17.24% of the 25% treated seeds were mixed with the 50% class. A similar trend was maintained in the YOLO11-n model, although the mixing rates were relatively lower and the diagonal values were higher.

These results reveal that visual changes do not occur linearly and sharply as the spraying rate increases, and that the boundaries between classes become blurred, especially at moderate levels (25–50%). Therefore, most of the error patterns stem from the inherent visual proximity of the classes rather than from model inadequacy.

This behavior is consistent with previous observations in seed treatment and coating studies, where intermediate treatment levels may exhibit heterogeneous and non-uniform surface coverage, making them visually less separable than untreated or fully treated seeds [1,3,11]. In particular, formulation-dependent adhesion behavior and variability in coating distribution may prevent intermediate dosage levels from producing sharply distinct visual patterns, which can directly affect image-based classification performance.

#### 4.3.2. Analysis of Misclassified Samples

**Figure 8:**
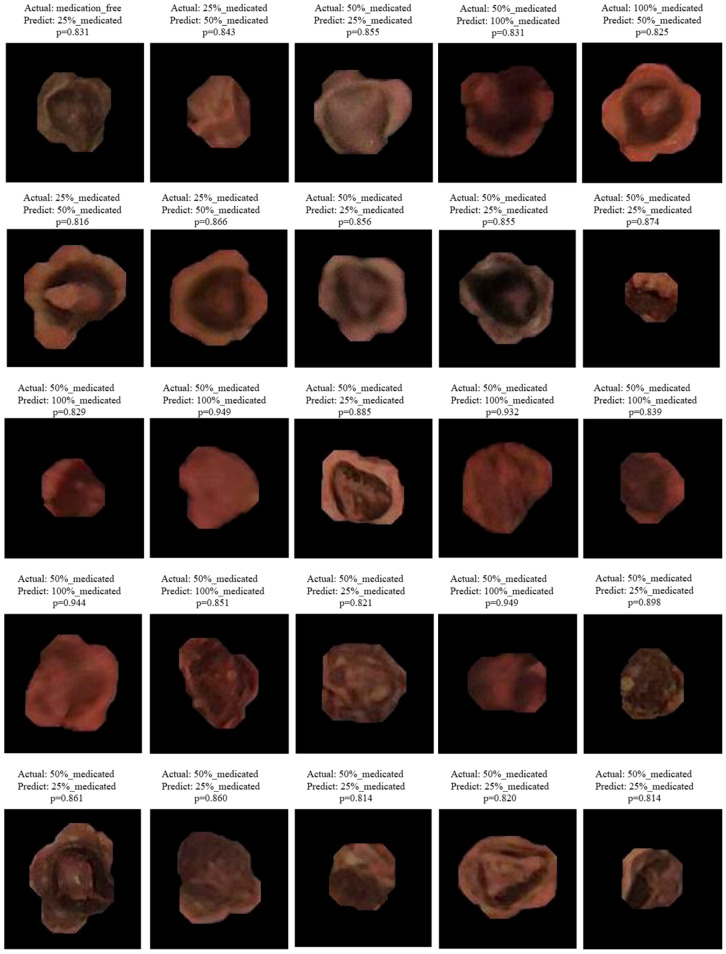
Selected misclassified examples for YOLOv8-n models.

**Figure 9:**
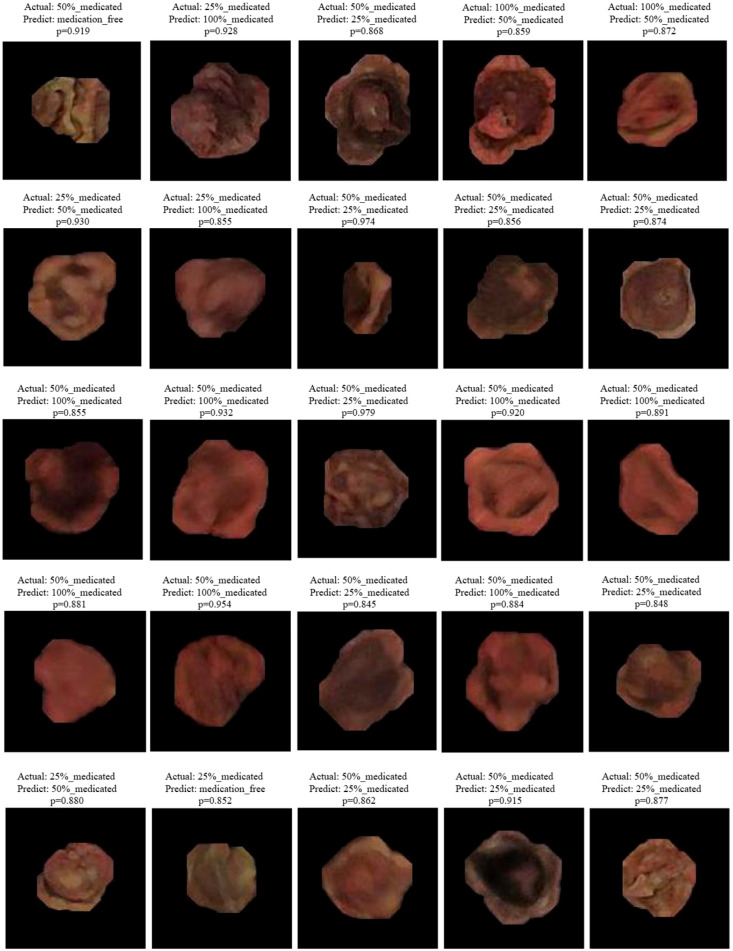
Selected misclassified examples for YOLO11-n models.

When misclassified samples were examined, it was observed that the majority of errors occurred between adjacent spraying levels. In particular, mixing between the 25–50% and 50–100% classes can be attributed to non-uniform pesticide distribution on the seed surface, local color intensities, and visual ambiguities caused by light reflections. Selected misclassified samples for the YOLOv8-n and YOLO11-n models are given in Figure 8 and Figure 9.

Visual analysis revealed that misclassifications largely occurred in seeds where the pesticide coating was not homogeneously distributed, exhibiting surface staining, irregular color transitions, or localized density differences. In such samples, even if the overall color of the seed belongs to the target class, the model’s dominant learning of local visual cues can lead to incorrect predictions. In the visual analysis of misclassified samples, the *p*-value assigned to each image represents the probability (confidence score) of the class predicted by the model. This value is presented as an interpretive measure, showing the degree of confidence with which the model determined the relevant class despite the incorrect prediction. Misclassifications with high *p*-values indicate that the model experiences instability, especially between visually similar pesticide application levels.

While the YOLOv8-n model shows that misclassifications are more concentrated between the 25% and 50% and 50% and 100% classes, the YOLO11-n model shows a relatively smaller percentage of these confounding factors and produces more consistent predictions. In particular, the YOLO11-n model has been observed to make more balanced decisions even in samples with partial surface spraying or irregular distribution.

These findings demonstrate that the YOLO11 architecture is capable of learning deeper and more effective feature representations, suggesting a potential advantage in discriminating subtle visual differences. However, the misclassifications observed in both models reveal that absolute visual distinctions between classes are not always possible due to the physical nature of the spraying process.

### 4.4. ROC-AUC Analysis and Model Discrimination

Evaluating classification performance solely using singular metrics such as accuracy or F1-score may not fully reflect the model’s discrimination capacity independent of decision thresholds. Therefore, in this study, the inter-class discrimination power of the selected YOLOv8-CLS and YOLO11-CLS models was also analyzed using ROC–AUC curves.

**Figure 10:**
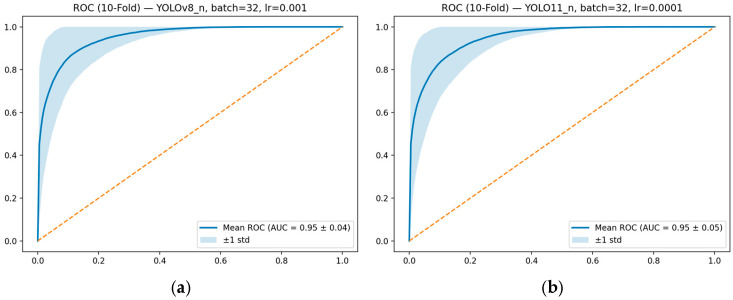
(**a**) Mean ROC–AUC curve obtained after 10-fold cross-validation for YOLOv8-n (batch = 32, lr = 0.001). (**b**) Mean ROC–AUC curve obtained after 10-fold cross-validation for YOLO11-n (batch = 32, lr = 0.0001).

Figure 10 presents the average of the ROC curves obtained across 10-fold cross-validation for both models. The ROC curves show the relationship between the true positive rate and the false positive rate at different decision thresholds. The AUC value, on the other hand, expresses how well the model can distinguish between classes as a threshold-independent measure.

The average ROC-AUC value obtained for the YOLOv8-n model was calculated as 0.95 ± 0.04. Similarly, the ROC-AUC value for the YOLO11-n model was found to be 0.95 ± 0.05. In both models, it is observed that the ROC curves deviate significantly from the diagonal random classification line and the curves are close to the upper-left corner. This indicates that the models can perform inter-class differentiation with high accuracy.

## 5. Discussion

The experimental results obtained in this study demonstrate that RGB images and deep learning-based classification approaches offer a feasible and reliable solution for automatically distinguishing pesticide application levels in sugar beet seeds. In particular, systematic evaluation of different scale variants of the YOLOv8-CLS and YOLO11-CLS architectures clearly revealed the impact of model capacity and architectural updates on classification performance.

In terms of overall performance, both architectures produce satisfactory accuracy and weighted F1-score values at all scales. However, while exhibiting similar accuracy levels to YOLOv8-CLS, YOLO11-CLS models were observed to offer more balanced precision-recall profiles and relatively more stable probability estimates (lower log-loss) in some configurations. This suggests that the YOLO11 architecture’s more up-to-date representation learning capacity enables it to create more stable decision boundaries, especially in scenarios where inter-class visual differences are limited. The high levels of ROC-AUC values in both architectures suggest that the models generally learn class distinction strongly, while the differences in accuracy and F1-score stem from uncertainties that arise in examples close to decision thresholds [6,7].

When the effect of model scale was examined, it was observed that while very small-scale (n) variants offered lower computational costs, classification performance became more stable in medium and large-scale (m–l–x) models. Particularly noteworthy was that at visually similar spraying levels (25% and 50%), models with higher representational capacity relatively reduced error rates. However, it was also observed that the performance improvement remained limited as the scale increased, and in some cases, saturation was reached. This finding highlights the importance of selecting not only the largest model but also the scale with the best balance between accuracy and computational cost for practical applications [1,3,11].

Class-based analyses and confusion matrices reveal that misclassifications are largely concentrated between intermediate dose levels (25% and 50%). This can be explained by the inherently nonlinear and heterogeneous nature of the spraying process. Even among seeds with the same nominal spraying rate, significant variations can occur in terms of coating thickness, color intensity, and surface distribution. Therefore, intermediate levels like 25% and 50% exhibit a transitional distribution rather than being visually separated by sharp boundaries. This presents a challenge to the model’s decision limits [1,3,11].

Visual inspection of the misclassified samples and their confidence values indicates that the model’s errors largely stem from ambiguous or borderline samples. In some samples, the model’s high-confidence decision to classify incorrectly points to situations where the visual characteristics of intermediate dose levels converge to upper or lower doses. This finding reveals that the problem is closely related not only to the model’s capacity but also to the physical and visual nature of the spraying process.

A review of the literature reveals that seed quality and defect detection problems are mostly addressed using either high-resolution but costly equipment, such as hyperspectral or multispectral imaging, or YOLO-based approaches primarily focused on object detection. The findings of this study demonstrate that a subtle and practically critical quality indicator like spraying rate can be differentiated with significant accuracy using only RGB images and classification-based YOLO-CLS architectures. In this respect, the study offers a more accessible alternative in terms of both equipment cost and data preparation process [13,17].

The results show that pesticide application levels in sugar beet seeds can be monitored image-based, and this approach shows potential for supporting future in-line monitoring or early warning systems. However, the fact that the model performance reaches a saturation point in scenarios where inter-class visual transitions are unavoidable suggests that this problem could be addressed in the future with different data representations or multimodal approaches.

Although the present study focuses on evaluating classification performance under controlled acquisition conditions, additional preprocessing strategies may further improve robustness in practical applications. Techniques such as color normalization, reflection suppression, or illumination balancing could help reduce visual variability caused by coating reflections or uneven color distribution. Investigating such targeted mitigation strategies would require a dedicated experimental design and therefore falls outside the scope of the present comparative study, but represents an important direction for future research.

In addition, the objective of this study was to conduct a controlled comparison between two successive generations of YOLO-based classification architectures within the same experimental framework. While comparisons with models from different families, such as lightweight CNN or transformer-based classifiers, could provide further insights into architectural trade-offs, including such models would substantially broaden the scope of the present work. Exploring cross-family model comparisons, therefore, represents another promising direction for future studies.

## 6. Limitations

The findings of this study demonstrate that the proposed approach is effective in classifying sugar beet seeds according to their treatment levels; however, some limitations should be considered. First, the dataset used consists of RGB images obtained under controlled laboratory conditions with constant lighting and camera configuration. This limits direct inferences about how the model performance would be affected under field conditions with variable lighting, different camera types, or background complexity.

Secondly, the classification was performed only at the image level, and the spatial homogeneity of pesticide distribution on the seeds or local density differences were not directly analyzed. In particular, high visual similarities between classes at intermediate application levels, such as 25% and 50%, can lead to the model producing inconsistent predictions in some examples.

Finally, this study used only RGB images; richer spectral representations, such as multispectral or hyperspectral information, were not included in the evaluation. This resulted in the inability to examine the impact of methods with higher discrimination potential, particularly for classes that are difficult to distinguish visually, within the scope of this study.

## 7. Future Work

The results obtained in this study demonstrate that it is possible to classify sugar beet seeds according to their treatment levels using RGB images. In future studies, evaluating the performance of the proposed approach in different shooting conditions and field environments is a key research direction. In particular, examining the model performance under varying lighting conditions, different camera types, and industrial-scale high-speed imaging systems will more comprehensively reveal the practical applicability of the method.

Another important research direction is extending the classification problem to finer granular levels. Although the current study addresses spraying levels under four discrete classes (0%, 25%, 50%, and 100%), classification with more intermediate levels or continuous dose estimation (regression-based approaches) could be evaluated in the future. Such approaches could contribute to the development of early warning and quality control mechanisms, particularly in spraying processes.

Furthermore, integrating complementary data sources such as multispectral or near-infrared (NIR) imaging, beyond just RGB images, is a promising direction for future studies. Such multi-modal approaches could allow for enhanced differentiation between visually close spraying levels.

Finally, integrating the proposed model into seed treatment lines as an online or semi-real-time decision support system represents a significant future step for industrial applications. Such a system could increase efficiency and reliability in production processes by detecting potential dose deviations early in the treatment process without requiring chemical analysis.

## 8. Conclusions

This study addresses the problem of classifying sugar beet seeds according to their treatment levels (0%, 25%, 50%, and 100%), and for this purpose, different scale variants of the YOLOv8-CLS and YOLO11-CLS architectures were comprehensively evaluated. Experiments performed on RGB images obtained under controlled conditions showed that YOLO-CLS approaches, which do not require bounding box annotation, offer an effective and feasible solution for image-level classification scenarios.

The results revealed that model scale plays a decisive role in classification success, and that medium-to-large scale variants, in particular, exhibited more stable performance in differentiating visually close spraying levels. The YOLOv8-CLS and YOLO11-CLS architectures generally produced similar accuracy levels; however, more stable decision profiles were obtained in some configurations. The observation of high ROC-AUC values in all models supports the strong learning of interclass differentiation.

Overall, this study demonstrates that pesticide application levels in sugar beet seeds can be automatically determined using only RGB images, without the need for additional chemical analyses. The presented approach provides a promising basis for developing future early warning and quality control mechanisms in seed treatment processes, particularly when integrated with appropriate hardware systems.

## Figures and Tables

**Table 1:** Classification performance of YOLOv8-CLS models under different scale variants, batch sizes, and learning rates (mean results over stratified 10-fold cross-validation).

	Batch Size	Learning Rate	Accuracy	Precision	Recall	F1-Score	Log_loss	ROC_AUC
**YOLOv8_n**	16	0.001	0.8208	0.818	0.8208	0.8176	0.4177	0.952
	16	0.0001	0.8243	0.8205	0.8243	0.8211	0.4211	0.9529
	32	0.001	0.8253	0.8233	0.8253	0.8232	0.4144	0.9531
	32	0.0001	0.826	0.8217	0.826	0.8222	0.4186	0.953
**YOLOv8_s**	16	0.001	0.8111	0.8078	0.8111	0.8063	0.4482	0.9476
	16	0.0001	0.8166	0.8128	0.8166	0.8127	0.4454	0.9483
	32	0.001	0.8151	0.8117	0.8151	0.81	0.4382	0.9485
	32	0.0001	0.8152	0.8113	0.8152	0.8114	0.4457	0.9481
**YOLOv8_m**	16	0.001	0.7859	0.7833	0.7859	0.7813	0.4937	0.9365
	16	0.0001	0.7884	0.7825	0.7884	0.783	0.4987	0.9373
	32	0.001	0.789	0.7859	0.789	0.7849	0.4869	0.9378
	32	0.0001	0.7892	0.7838	0.7892	0.7846	0.4943	0.9376
**YOLOv8_l**	16	0.001	0.7954	0.7922	0.7954	0.791	0.4813	0.9404
	16	0.0001	0.7956	0.7909	0.7956	0.7907	0.4871	0.9411
	32	0.001	0.7974	0.7929	0.7974	0.7926	0.4679	0.9424
	32	0.0001	0.8004	0.7963	0.8004	0.7967	0.4763	0.9425
**YOLOv8_x**	16	0.001	0.7849	0.7791	0.7849	0.7763	0.4969	0.9362
	16	0.0001	0.7901	0.7844	0.7901	0.7837	0.5006	0.9371
	32	0.001	0.7893	0.7863	0.7893	0.786	0.4861	0.9375
	32	0.0001	0.7902	0.7857	0.7902	0.7867	0.4934	0.9378

**Table 2:** Classification performance of YOLO11-CLS models under different scale variants, batch sizes, and learning rates (mean results over stratified 10-fold cross-validation).

	Batch Size	Learning Rate	Accuracy	Precision	Recall	F1-Score	Log_loss	ROC_AUC
**YOLO11_n**	16	0.001	0.8048	0.7995	0.8048	0.7992	0.4458	0.9463
	16	0.0001	0.813	0.8085	0.813	0.8081	0.4464	0.9473
	32	0.001	0.8112	0.8088	0.8112	0.8076	0.4388	0.9473
	32	0.0001	0.8178	0.8137	0.8178	0.8135	0.4277	0.9495
**YOLO11_s**	16	0.001	0.8011	0.7959	0.8011	0.7953	0.4594	0.9434
	16	0.0001	0.8074	0.8026	0.8074	0.8009	0.4504	0.9462
	32	0.001	0.8068	0.8041	0.8068	0.8038	0.4466	0.9457
	32	0.0001	0.81	0.8052	0.81	0.8062	0.4417	0.9468
**YOLO11_m**	16	0.001	0.7907	0.7855	0.7907	0.7845	0.481	0.9386
	16	0.0001	0.7964	0.7904	0.7964	0.789	0.4789	0.9396
	32	0.001	0.7963	0.7919	0.7963	0.7912	0.4687	0.9403
	32	0.0001	0.7999	0.7958	0.7999	0.7958	0.4656	0.9411
**YOLO11_l**	16	0.001	0.7992	0.7959	0.7992	0.7946	0.4616	0.9431
	16	0.0001	0.8051	0.8004	0.8051	0.8	0.4576	0.9442
	32	0.001	0.8051	0.8008	0.8051	0.7991	0.4525	0.9441
	32	0.0001	0.8071	0.8022	0.8071	0.802	0.4505	0.9449
**YOLO11_x**	16	0.001	0.7987	0.7958	0.7987	0.7915	0.476	0.9413
	16	0.0001	0.8059	0.8009	0.8059	0.7993	0.4578	0.945
	32	0.001	0.8046	0.8029	0.8046	0.8006	0.4565	0.9441
	32	0.0001	0.808	0.8037	0.808	0.804	0.4529	0.9451

## Data Availability

The dataset can be accessed through the link provided. http://citedata.com/Sugar_Beet_Seed_Images_Dataset.zip (accessed on 15 February 2026).

## Abbreviations

The following abbreviations are used in this manuscript:

YOLO	You Only Look Once
KNN	K-Nearest Neighbors
ML	Machine Learning
PCA	Principal Component Analysis
LDA	Linear Discriminant Analysis
SVM	Support Vector Machine
CNN	Convolutional Neural Network
CLS	Classification
GB	Giga Byte
mAP	Mean Average Precision
TP	True Positive
FP	False Positive
FN	False Negative
TN	True Negative
ROC-AUC	Receiver Operating Characteristic and Area Under the Curve
